# Suture of Wounds in Time of Attack

**Published:** 1918-03

**Authors:** 


***Suture of Wounds in Time of Attack.*** By MARQUIS, DESCAZAL, LUQUET, and MORLOT. Trans. and Abs. from the *Bulletins et Mémoires de la Société de Chirurgie de Paris*, Dec. 25, 1917.

The authors describe their efforts to procure for every wounded man during periods of attack " the benefit of suture whenever the circumstances permitted. "

For this purpose their ambulance (Auto-Chir. 22) was provided with a large operative tent, designed by themselves, and equipped with every possible facility. It was provided with 6 operating rooms and manned by 11 surgical and 4 radiographical teams. During the first 24 hours of the attack they were able to operate upon 178 of the wounded. Every possible provision was made for sterilization and for speedy evacuation. Patients were received within from 6 to 12 hours after injury. Arrangements were made whereby all light cases and those for whom there was little chance of life, should be treated elsewhere.

In spite of all these precautions the obstacles to suture were very great. The influx of the wounded was very sudden, amounting in one day to 1,139 cases. During such a time the length of an operation for suture is a serious hindrance. The necessity of retaining cases that have been sutured is a further complication.

To overcome such obstacles the following measures are taken:

1. Slight wounds of the soft parts, when single, are evacuated immediately after excision to the base hospitals for a delayed primary suture.

2. All wounds in which primary suture is imperative, for functional or vital reasons, should, when conditions permit, be sutured at once.

3. The larger part of the wounds of soft parts may be held for secondary suture on the days following the attack.

Those in a condition of shock, with a blood pressure, according to Pachon, of less than 10 cm., are sent at once to a warming chamber. This is a room 8 meters long and 2.25 meters wide. It contains five beds, with the forward legs raised in such a manner as to bring the patient's head considerably below the level of his feet. The temperature in this room is maintained at from 40° to 50° C. Out of 21 patients placed in this room during an attack, 6, whose pressure, according to Pachon, was hardly perceptible, died. The remaining 15, with pressures varying from 5 to 9 cm., were soon sufficiently restored to undergo operation. They had also been given a massive injection of adrenaline serum.

Out of a total of '442 wounded, 298 patients (with 550 wounds) could not be evacuated. Of the wounds of the soft parts, 53 were given primary suture; 257, secondary suture; a total of 308 sutures or 90 o/o.

Of the 133 bone wounds, 26 were given primary suture and 77 secondary suture; a total of 105 sutures or 77 o/o.

Of 34 joint wounds, 24 were given primary suture (70 o/o). The authors remark that the wounds of the joints seemed to profit most by primary suture.

Good results were obtained also in wounds of the skull, the abdomen, and the thorax.

For all the cases, the mortality was 21 or 4.7 o/o.

The authors would not insist that suture be practised invariably in time of attack, but they believe that, whenever operative facilities and hospital accommodation permit, every patient should be given the benefit of primary suture.

